# On the Road to Immunotherapy—Prospects for Treating Head and Neck Cancers With Checkpoint Inhibitor Antibodies

**DOI:** 10.3389/fimmu.2018.02182

**Published:** 2018-09-24

**Authors:** Frank J. Ward, Lekh N. Dahal, Rasha Abu-Eid

**Affiliations:** ^1^Institute of Medical Sciences, School of Medicine, Medical Sciences and Nutrition, University of Aberdeen, Foresterhill, Aberdeen, United Kingdom; ^2^Centre for Cancer Immunology, Faculty of Medicine, University of Southampton, Southampton General Hospital, Southampton, United Kingdom; ^3^Institute of Dentistry, School of Medicine, Medical Sciences and Nutrition, University of Aberdeen, Foresterhill, Aberdeen, United Kingdom

**Keywords:** head and neck squamous cell carcinoma, checkpoint inhibitor, immunotherapy, T cells, PD-1

## Abstract

Head and neck cancers (HNC) represent a heterogeneous cluster of aggressive malignancies that account for 3% of all cancer cases in the UK. HNC is increasing in frequency particularly in the developing world, which is related to changes in risk factors. Unfortunately, the mortality rate is high, which is chiefly attributed to late diagnosis at stages where traditional treatments fail. Cancer immunotherapy has achieved great successes in anti-tumor therapy. Checkpoint inhibitor (CI) antibodies enhance anti-tumor activity by blocking inhibitory receptors to drive tumor-specific T and NK cell effector responses. Since their introduction in 2011, CI antibodies have been approved for many cancer types including HNC. Here, we examine the development of CI therapies and look forward to future developments for treatment of HNC with CI therapies.

## Introduction

The notion of boosting anti-tumor immunity as a means of treating cancer has been escalated by the recent unveiling of exciting new immunotherapies including the checkpoint inhibitor (CI) antibodies (1). CI antibodies selectively activate adaptive immunity to locate and obliterate tumors anywhere in the body and can also generate an enduring disease remission (2). The recent approval of six CI antibody therapies for treating a range of cancers (3–8) heralds a golden age of immunotherapy, with the promise of further novel, better immune-boosting technologies and combination treatment strategies to come (9). But there are also caveats such as poor patient response frequency, the potential for serious immune related side effects and generally a lack of biomarkers which can guide the use of these therapies (10). In this review, we reflect on the developing prospects for CI therapy to treat head and neck cancers (HNC).

## Head and neck cancers

Head and neck cancers, of which the majority are squamous cell carcinomas (HNSCC), represent a collection of neoplasms that are difficult to treat and whose incidence in the UK and worldwide has increased by around 30% since 1990. In 2015, 12,000 individuals were diagnosed with HNC in the UK [CRUK, oral cancer statistics, 2018], representing 3% of all cancers. Annually, there is an estimated 600,000 cases worldwide, which affect the oral cavity, oropharyngeal, hypopharyngeal, and laryngeal tissues (11, 12). Increased incidence is associated with known risk factors including high use of both tobacco and alcohol. In certain parts of the world, in particular South East Asia, the incidence of HNC is much higher and is reported as high as 30% of all cancers in India, with the major risk factor being betel quid (pan) chewing in all its forms, which almost invariably includes tobacco (smokeless tobacco) (11, 12). Many cases of HNC are also associated with infection by human papillomavirus (HPV) strains 16 and 18, well established high-risk viral types in other malignancies, most notably cervical cancer (13). Patients with HPV^+^ tumors, however, have a better outcome in terms of both survival and reduced risk of recurrence compared with HNC in which no virus can be detected (13). This latter observation may reflect a greater intrinsic immunogenicity associated with HPV infection and this perception is supported by immune profiling studies that find increased effector T cell infiltrates in HPV^+^ compared with HPV^−^ tumors (14).

The current treatment standard of care for HNSCC is to treat recurring or metastatic tumors with cetuximab, an anti-epidermal growth factor receptor antibody, together with platinum based cis- or carboplatin chemotherapy plus 5-flurouracil and methotrexate, which is further supported where appropriate by surgery and radiotherapy (15), and in some instances augmented by the taxanes, docetaxel and paclitaxel.

In 2016, two anti-PD-1 checkpoint inhibitor monoclonal antibodies (mAbs), pembrolizumab and nivolumab provided new options for cisplatin resistant recurring or metastatic HNSCC following accelerated FDA approval based on encouraging clinical trial data (16, 17) and precedence of response efficacy in large phase III clinical trials of melanoma and non-small cell lung cancer in which both antibodies had already demonstrated significant improvements in patient outcomes compared to current standard of care therapy (4, 5, 18, 19). This has led to further clinical trials for HNSCC with larger patient cohorts primarily with the aim of comparing anti-PD-1 antibodies alone or together with current platinum-based therapies and cetuximab. Currently, there are more than 90 clinical trials involving established CI inhibitor therapies and HNSCC.

## Immune checkpoints

The immune system is a decision-making entity which, when not required remains quiescent but vigilant for the emergence of a new pathogenic challenge. Once that challenge arrives, the immune system ramps up immune processes shaped to deal specifically with each new pathogenic threat, powerful enough to clear the pathogen, but which may also carry some risk of bystander damage to host cells and tissues. After pathogen clearance, the immune system also needs to return to its former quiescent state to avoid any further damage. Immune checkpoints, primarily receptors on immune cells, regulate both immune response intensity to prevent host tissue damage, and also resolve the immune response after pathogen clearance (20). Interactions between checkpoint receptors and their ligands can be restricted to immune cell subsets but can also take place between immune and non-immune cells (21). Environmental cues within an inflammatory lesion up-regulate expression of receptors on non-hematopoietic and non-lymphoidal cells such as epithelia that then engage with immune effector cells to suppress and eventually quell their activity (22, 23).

There are several immune checkpoint receptors, all of which have individual expression patterns on a variety of immune cells and, therefore, contribute to immunoregulation at different levels. Perhaps the best known and most fundamental checkpoint receptor is CTLA-4 (CD152) (24), which plays a role both in the priming of naïve T cells and also control of effector T cell response intensity (25–27). Other checkpoints include PD-1 (CD279) (28, 29), ICOS (CD278) (30), 4-1BB (CD137) (31), OX40 (CD134) (32), LAG-3 (CD223) (33), TIM-3 (34), TIGIT (35), VISTA (36), BTLA (CD272) (37), and GITR (38), which display a hierarchy of expression on different cell types and therefore exert a more selective control over interactions both between immune cell subsets and between immune and non-immune cells (39). Analyses of immunogenic HNSCC, suggest that in many individuals the tumors appear “primed” to make potent anti-tumor effector T cell responses and can therefore be considered suitable for CI therapy. There is an urgent need, therefore, to develop genetic and histological response biomarkers that efficiently identify and stratify responsive patient cohorts to one or more of these CI therapies, together with other therapies designed to increase tumor immunogenicity (40, 41).

## Checkpoint inhibitor antibodies

Checkpoint inhibitor (CI) antibodies target immune cell checkpoint receptors to selectively activate antigen-specific anti-tumor T cell responses. In 2013, CI therapies together with CAR-T cell immunotherapy were considered to be the most important scientific breakthrough of the year by *Science* (42). The efficacy of CI therapies has been ground-breaking in the treatment of melanoma, non-small cell lung cancer (NSCLC) and other cancers, including HNSCC, offering clear advances over other established chemo- and immunotherapies in terms of patient response frequency, as well as efficacy and durability of response. In general, tumor immunogenicity is considered to be the most important factor in determining whether or not a particular type of cancer will respond to CI therapy (43), but this has not prevented CI therapies from being tested in most types of cancer in ongoing clinical trials and it will be some time before it becomes apparent which cancers are most responsive to this type of CI immunotherapy.

**Figure 1:**
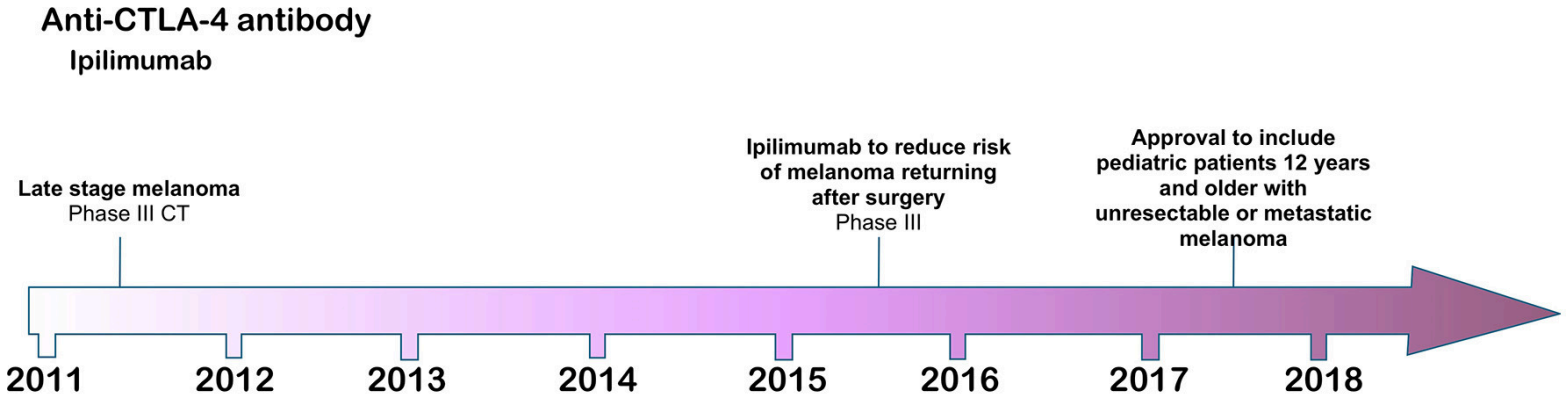
Time line of FDA approvals for ipilimumab (July 2018).

**Figure 2:**
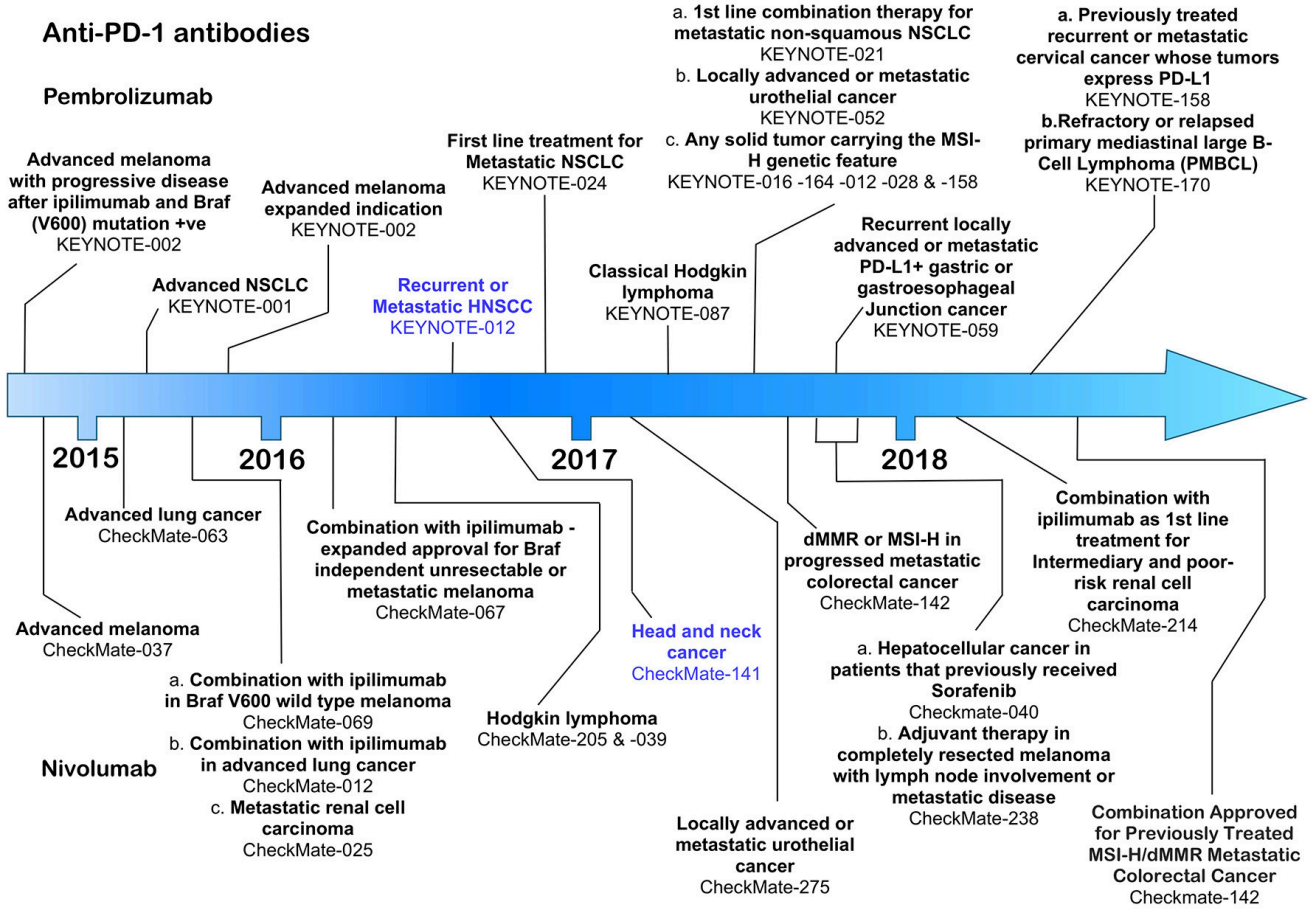
Time line of FDA approvals for the anti-PD-1 antibodies pembrolizumab and nivolumab (July 2018).

**Figure 3:**
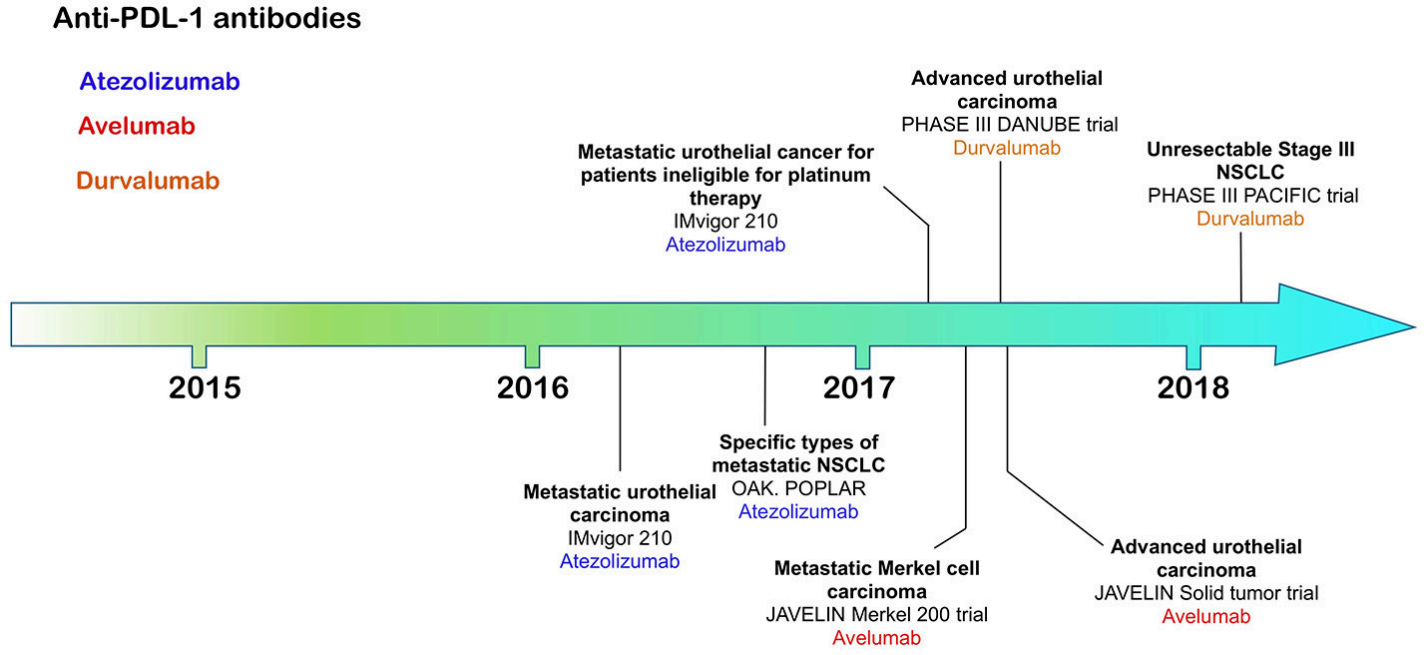
Time line of FDA approvals for anti-PD-L1 antibodies Atezolizumab, Avelumab, and Durvalumab (July 2018).

The six CI antibodies with FDA approval so far have specificities for CTLA-4 (ipilimumab), PD-1 (pembrolizumab, nivolumab) and PD-L1 (atezolizumab, durvalumab, and avelumab) checkpoint receptors. Collectively, they have been approved for advanced melanoma, non-small cell lung cancer, renal cell carcinoma, urothelial and bladder cancer, HNSCC, metastatic Merkel cell carcinoma, refractory classical Hodgkin lymphoma and gastric cancer (Figures 1–3). Another CTLA-4 antibody, tremelimumab, is in advanced stages of clinical trials, while cemiplimab an anti-PD-1 IgG_4_ antibody is likely to be FDA approved soon for the treatment of advanced cutaneous squamous cell carcinoma. Many more CI therapies are under development (44), and by 2025, the checkpoint inhibitor market is expected to exceed $40 billion worldwide.

## CTLA-4

The first checkpoint inhibitor antibody, ipilimumab, was approved by the FDA in 2011 for the treatment of metastatic melanoma (3) based on a phase III clinical trial in which the antibody significantly extended melanoma patient survival compared with standard of care therapy. The CTLA-4 (CD152) target of ipilimumab is an inhibitory regulator of T cell costimulation (45, 46), modulating the priming and activation of naïve T cells, as well as the intensity and potency of both CD4^+^ T helper and CD8^+^ cytotoxic effector T cell responses. CTLA-4 is an inhibitory counterpart to the stimulatory CD28 receptor on T cells, which is an essential component of antigen-specific naïve T cell costimulation during initial priming by dendritic cells (DC). Like CD28, CTLA-4 on T cells interacts with the B7.1/B7.2 (CD80/CD86) ligands on DC, but with higher affinity to out-compete its costimulatory partner and thus to inhibit T cell activation (47). As a CI therapy, therefore, antibody blockade of CTLA-4 functions at a fundamental level to restore effective DC priming of naïve T cells, inducing full activation and expansion of nascent effector T cell populations against tumor neoantigens (48).

The non-redundant function of CTLA-4 in regulating immune cell homeostasis was demonstrated by CTLA-4 knockout mice, which die soon after birth from massive lymphocytic infiltration of tissues and organs (49, 50). Healthy homeostasis in these mice could be restored, however, by infusion of recombinant soluble CTLA4-Ig (51) or by generating chimeric mice in which CTLA-4^+^ T cells are able to regulate CTLA-4^−^ T cells to prevent their unregulated expansion (52, 53). In humans, analyses of heterozygous CTLA-4 gene haploinsufficiencies, or reduced expression of CTLA-4 caused by mutation in the LRBA gene (encoding the lipopolysaccharide-responsive and beige-like anchor protein), which is thought to regulate CTLA-4 protein turnover (54), have also revealed complex pathological phenotypes that correspond to unregulated T cell responses, including Treg dysfunction, effector T cell hyper-proliferation, non-lymphoid organ infiltration and autoantibody production (55, 56). Patients with these pathologies also respond well to abatacept, the human recombinant soluble CTLA4-Ig. These observations demonstrate that CTLA-4 can be used by immune cells to extrinsically regulate effector T cell populations. CTLA-4 is constitutively expressed in higher amounts on regulatory T cells (Treg) and is essential for their immunoregulatory function (57), although how these cells utilize CTLA-4 to control effector T cell populations still needs to be fully elucidated (58).

The therapeutic potential of CTLA-4 antibody blockade was first demonstrated in murine cancer models of melanoma, mammary and prostate cancer (59–61). In the B16-BL6 and B16-F10 murine models of solid and metastatic melanoma, in particular, anti-CTLA-4 antibody blockade of T cells alone was not effective in eliminating tumors, and their potential for driving anti-tumor immunity was revealed only after treatment of the mice with a granulocyte/macrophage colony-stimulating factor (GM-CSF)-expressing tumor cell vaccine, which enhanced dendritic cell activity and also enhanced the generation of B16 melanoma-specific T cells (60). This increase in immunogenicity provided the antigen required by tumor specific effector T cells, allowing complete dissolution of the tumors. These experiments in mice shaped the future therapeutic strategy for CI blockade in humans and it also highlighted the requirement for tumor immunogenicity as an essential requirement for successful treatment. These early experiments in CI therapy highlighted the importance of the mechanisms and environmental cues that drive the release and display of tumor associated and tumor specific neoantigens (62).

Ipilimumab was approved by the FDA in 2011 for use in metastatic melanoma refractory to conventional treatments, in which patients receiving ipilimumab with or without the melanoma-derived gp100 tumor-associated antigenic peptide co-vaccine demonstrated increased overall survival of 10 months compared with 6.4 months in patients receiving the vaccine alone (3). An interesting and important feature of ipilimumab CI therapy is that it can induce an enduring remission from melanoma disease in approximately 22% of metastatic melanoma patients receiving the therapy (2). Despite this success, ipilimumab has little beneficial effect for most patients receiving it and it can also provoke very severe immune related adverse events, including dermatitis, colitis, hypophysitis, and other inflammatory events (3). Further, there is currently no reliable biomarker with which to stratify patients by identifying those responsive to the therapy. Ipilimumab is generally administered four times over a period of approximately 3 months at a dose of 3 or 10 mg/kg body weight.

Despite the ground-breaking success of ipilimumab as the first CI therapy, there is still some controversy of how it is able to “release the brakes” of the immune system to drive anti-tumor immunity. Although the simple hypothesis is that CTLA-4 blockade generally inhibits CTLA-4 engagement with its B7 ligands, thereby allowing CD28 costimulation and full T cell activation to take place, there is some evidence that other factors may also be at play. Currently, the best accepted hypothesis is that anti-CTLA-4 antibodies mediate at least some of their immune boosting effects by engaging with activating Fc gamma receptors (FcγR) by binding to CTLA-4 on Treg to induce macrophage mediated depletion of the Treg through antibody-dependent cell-mediated cytotoxicity (ADCC) within the tumor environment or at the infusion site (63). This depletion would therefore allow reactivation of tumor infiltrating lymphocytes (TIL) to drive productive anti-tumor immunity. Other groups have provided evidence that anti-CTLA-4 antibodies can directly engage with CTLA-4^+^ tumor cells to drive ADCC (64, 65), but this is unlikely to fully explain the profound anti-tumor effects of CTLA-4 based CI therapy.

No CTLA-4 based CI therapy has been approved for the treatment of HNSCC but there are several clinical trials underway involving either ipilimumab or tremelimumab. Almost all of these are clinical trials involving combinations of anti-CTLA-4 mAbs either with other CI therapies, or current standard of care HNSCC therapies such as cisplatin and cetuximab.

## PD-1/PD-L1

Programmed death-1 (PD-1) is a checkpoint receptor primarily expressed by T cells that plays a crucial role in regulating and resolving adaptive effector T cell immune responses (28). PD-1 binds two ligands PD-L1 (CD274, B7-H1) (66) and PD-L2 (CD273, B7-DC) (67). PD-L1, has a very broad distribution on normal tissues and PD-1: PD-L1 interactions between immune and non-immune cells within inflammatory milieus are thought to maintain peripheral tolerance by suppressing effector T cell responses (68, 69). Induction of signaling through the PD-1 receptor suppresses IL-2 production in T cells and renders them less antigen-responsive (70). This anergic “exhaustion” phenotype is reversible by selective blockade of either PD-1 or PD-L1 (71). PD-L2 expression is less abundant and restricted mainly to professional antigen presenting cells but induces similar effects in T cells (72).

The therapeutic benefits of CTLA-4 antibody blockade were identified in murine tumor models, but the potential for PD-1 or PD-L1 blockade as a novel CI therapy arose from observational studies in humans. Soon after initial identification of PD-L1, analysis of renal cell carcinoma (RCC) patient survival outcomes following nephrectomy identified that patients with high expression levels of PD-L1 on either RCC tumor cells, RCC tumor infiltrating lymphocytes, or both, were at significantly increased risk of death from aggressive tumor progression (73). This important observation, together with extensive analyses of PD-L1 on tumor cell lines (67) and tumors including HNSCC (74), raised the notion of a novel immune evasion mechanism through which cancer cells nullify anti-tumor effector T cell responses by engaging PD-1 and inducing the exhaustion phenotype (75). These latter observations in HNSCC were further qualified by more recent studies in which analysis by PCR and immunohistochemistry of 41 esophagectomy tumors identified elevated levels of PD-L1 to be associated with a poor prognosis particularly in advanced tumors (76), while another study, however, did not find a clear correlation between tumor cell expression of PD-L1 and poor prognosis, but did identify elevated expression of PD-L1 on infiltrating immune cells, including T cells, macrophages and dendritic cells, to correlate significantly with increased overall survival (77). In addition, increased abundance of CD3^+^ and CD8^+^ T cell infiltrates also associated with prolonged survival outcomes (77).

With regard to HPV^+^ tumors, the PD-1:PD-L1 nexus may be especially relevant given that the effector T cell exhaustion phenotype, induced by engagement of PD-1 with PD-L1, is often associated with viral infection (70), and is likely a critical element in the induction of an artificial immune privileged microenvironment (78). HPV^+^ oropharyngeal tumors are associated with increased levels of T cell infiltrates and following conventional therapy overall survival and reoccurrence are both improved compared with HPV^−^ tumors (77, 79) suggesting that they are in effect primed for an anti-tumor response because of the anti-viral response. However, although HPV positive status signals a better outcome for HNSCC, recent studies investigating HPV integration into the host genome suggest that HPV integration into key gene sites including the PD-L1 gene may be a critical marker for patient outcome with reduced survival in patients with integration positive HPV tumors (13).

These studies have led to the rapid development of both anti-PD-1 and anti-PD-L1 checkpoint inhibitor therapies to block the PD-1: PD-L1 axis, which have since demonstrated significant improvements in patient outcomes in clinical trials for a range of cancers including HNC over the last five years.

Antibodies specific for the PD-1 receptor were the first CI therapies to be introduced after ipilimumab, initially for the treatment of melanoma, and have had much greater success than ipilimumab clinically and commercially. In 2017, sales of Keytruda™ (pembrolizumab) and Opdivo™ (nivolumab) were reported as $3.8 billion and $4.95 billion respectively with worldwide growth in sales from 2016 to 2017 of 171 and 31% respectively. Because CI therapies, including the PD-1 antibodies, target the immune system rather than the tumor, CI antibodies can theoretically be used to treat many forms of cancer and this notion has been successfully translated into the clinic with regard to anti-PD-1.

Unlike the view of anti-CTLA-4 antibodies in which binding FcγR may indirectly contribute to their therapeutic effects, anti-PD-1 mAbs are mechanistically straightforward and function simply by blocking engagement of PD-1 with its ligands PD-L1 and PD-L2. Indeed, engagement of FcγR was detrimental to their therapeutic potency (80) and thus most anti-PD-1 antibodies are of the IgG_4_ antibody subclass, which has weak binding associations with FcγR.

So far, the anti-PD-1 antibodies have been FDA approved for the treatment of advanced melanoma, non-small cell lung cancer, renal cell carcinoma, classical Hodgkin's lymphoma, urothelial cancer, gastric cancer and head and neck cancer (Figure 2). Throughout clinical trials, anti-PD-1 antibodies demonstrated an increase in response frequency in patients compared with current standard of care and were accompanied by lower risk and frequency of serious immune related adverse events compared with ipilimumab (81). Both pembrolizumab and nivolumab were first approved for use by the FDA after being granted an accelerated approval protocol in 2014 for the treatment of unresectable metastatic melanoma in patients carrying the V600 BRAF mutation (5, 82).

## Anti-PD-1 therapy in HNSCC

Pembrolizumab, a humanized IgG_4_ antibody was approved on August 5th, 2016 under the FDA's accelerated approval programme, based on data from the KEYNOTE-012 phase 1b clinical trial, which assessed the therapeutic effects of pembrolizumab in patients with HNSCC, triple negative breast cancer, gastric cancer and urothelial cancer (16). HNSCC patients whose disease had progressed following platinum-based therapy received pembrolizumab at either 10 mg/kg body weight every 2 weeks (*n* = 53) or a fixed dose of 200 mg every 3 weeks (*n* = 121) until disease progression or the development of intolerable toxicity (16). Patients received treatment for a maximum of 24 months. The overall response rate in the combined HNSCC patient cohorts was 16 with 5% of those achieving a complete response. The duration of response in 82% of the responsive patients lasted more than 6 months. Pembrolizumab has also shown clinically significant activity in patients with both HPV^+^ or HPV^−^ tumors (83). Among the immune related adverse events associated with therapy were pneumonitis, colitis, hepatitis, adrenal insufficiency, diabetes mellitus, and skin toxicities (16). Despite these promising results, however, pembrolizumab failed to meet its pre-specified primary endpoint of overall survival in the larger phase III KEYNOTE-040 clinical trial, which compared treatment with pembrolizumab at a fixed dose of 200 mg every 3 weeks with cetuximab, methotrexate (ESMO 2017 Press Release). Another phase III KEYNOTE trial (KEYNOTE-048, NCT02358031) is currently underway in which pembrolizumab alone (fixed 200 mg dose in 3 weekly cycles for up to 24 months), or pembrolizumab (fixed 200 mg dose) together with a platinum-based therapy plus 5-fluorouracil and compared with cetuximab combined with a platinum-based therapy and 5-flurouracil. The primary completion date for this trial is 31st December 2018. In addition, more than 50 clinical trials involving pembrolizumab in HNSCC are underway, most of which are focussed on therapies that combine radiotherapy or platinum-based therapies with pembrolizumab.

Nivolumab, a fully human IgG_4_ was also FDA approved in 2016 following completion of the CheckMate-141 open-label, phase III clinical trial in which 361 HNSCC patients with recurrent squamous-cell carcinoma of the head and neck whose disease had progressed within 6 months after platinum-based chemotherapy, were treated with nivolumab or standard therapy alone (17). Patients received nivolumab at a dose of 3 mg/kg every 2 weeks with the end point of overall survival as the critical marker of improvement over the standard of care therapy. Patients receiving nivolumab were compared at a 2:1 ratio with patients receiving post-platinum standard of care therapies including methotrexate, docetaxel and cetuximab (17). Patients receiving nivolumab had an overall survival median of 7.5 months [95% confidence interval [CI], 5.5 to 9.1] in the nivolumab group compared with 5.1 months (95% CI, 4.0 to 6.0) in the standard-therapy group (17). PD-L1 expression levels were examined in 72% of the 361 patients in the clinical study and 57.1% of those had PD-L1 expression levels of ≥ 1%. Individuals with PD-L1 levels ≥ 1% responded better to nivolumab compared with the patient cohort in which PD-L1 was less than 1% (17). Patients specifically with oropharyngeal HNSCC also responded better to nivolumab independent of their HPV status. This study also revealed that quality of life measures in patients receiving nivolumab remained stable or improved slightly, whereas patients receiving standard therapy suffered significant deterioration at 15 weeks after commencement of therapy (17). Toxicities included pneumonitis, dermatitis, and endocrine dysfunction, although serious adverse events were significantly lower in the nivolumab compared with standard care study arm (17). As for pembrolizumab, nivolumab is currently the subject of several further clinical trials, primarily in which it is paired with other treatment options including anti-CTLA-4 ipilimumab (84).

## Anti-PD-L1 antibodies

The most recently FDA approved CI antibodies are three anti-PD-L1 antibodies—atezolizumab, avelumab and durvalumab; approved for urothelial/bladder cancer (6–8), non-small cell lung cancer (atezolizumab, durvalumab) and Merkel cell carcinoma (avelumab). All of these therapies rely on expression of PD-L1 on the target tumor, allowing both patient stratification through increased response frequency based on tumor expression levels of PD-L1.

Atezolizumab, a humanized IgG_1_ antibody engineered to reduce any potential for ADCC or CDC, was the first anti-PD-L1 antibody to be approved for use in advanced urothelial carcinoma patients whose disease had worsened after a platinum-based therapy (6). Bladder tumors have relatively high expression levels of PD-L1 compared with other tumors identifying this type of cancer as a suitable target for anti-PD-L1 antibodies (85). Patients in this phase II clinical trial were segregated according to tumor-infiltrating immune cell and tumor levels of PD-L1 expression by immunohistochemistry using the Ventana SP142 assay (6). Immune cell (IC) PD-L1 status was grouped into IC0 (<1%), IC1 (≥1% but <5%) and IC2/3 (≥5%). Over 26% of patients with PD-L1 positive tumor TIL experienced an anti-tumor response compared with 9.5% negative for PD-L1 supporting need for PD-L1 screening.

Avelumab, a fully human IgG_1_ antibody with *retained* potential to induce ADCC, was first approved for the treatment of Merkel cell carcinoma (MCC) (7), an aggressive cancer associated with polyomavirus infection with poor prognosis, ineffective chemotherapeutic options and low survival compared with other skin cancers. Avelumab therapy increased significantly overall survival, progression-free survival and durability of response compared with chemotherapy (7). The efficacy of avelumab was independent of PD-L1 tumor expression or polyomavirus infection (7).

Durvalumab, a fully human IgG1 engineered to reduce ADCC or CDC, was first approved for the treatment of urothelial carcinoma followed by approval for stage III unresectable NSCLC (8, 86).

For HNSCC, all three anti-PD-L1 antibodies are currently in clinical trials and in nearly all cases they are being combined with other experimental or established therapies.

## Combination therapy

**Figure 4:**
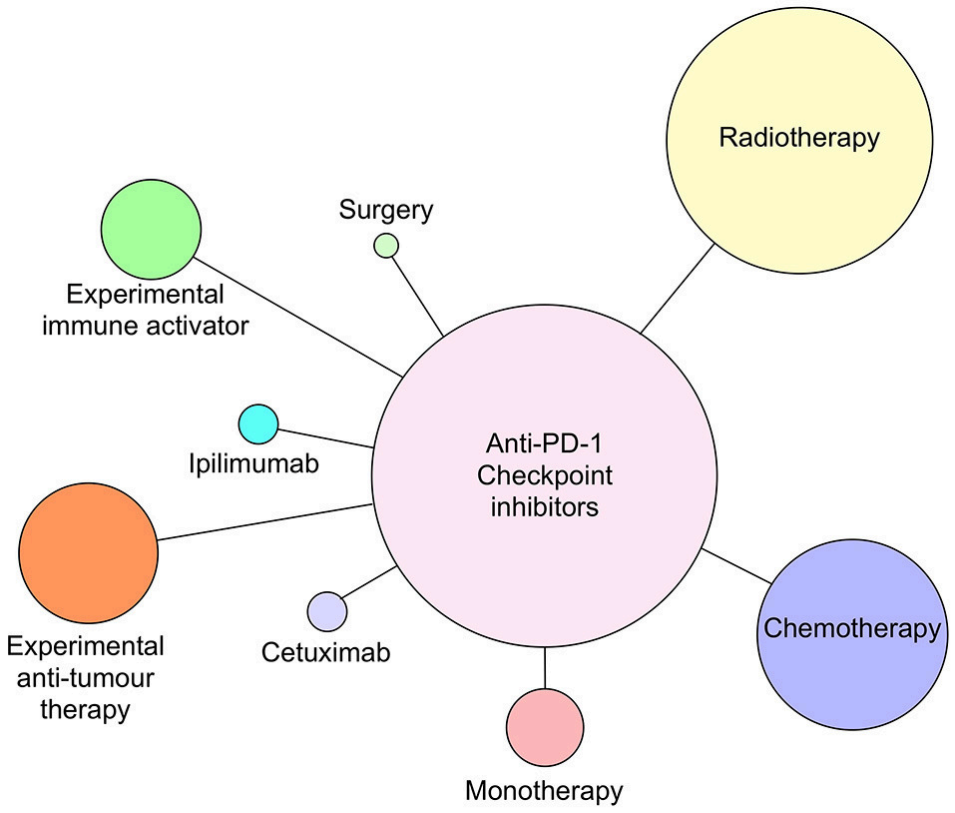
Anti-PD-1 antibodies pembrolizumab and nivolumab currently in clinical trials as monotherapy or combined with radiotherapy, chemotherapy, cetuximab, ipilimumab (anti-CTLA-4), surgery, or novel immune activating or tumor suppressive therapies as of July 2018. Circle size reflects relative number of times each type of therapy has been combined with anti-PD-1 therapy.

**Figure 5:**
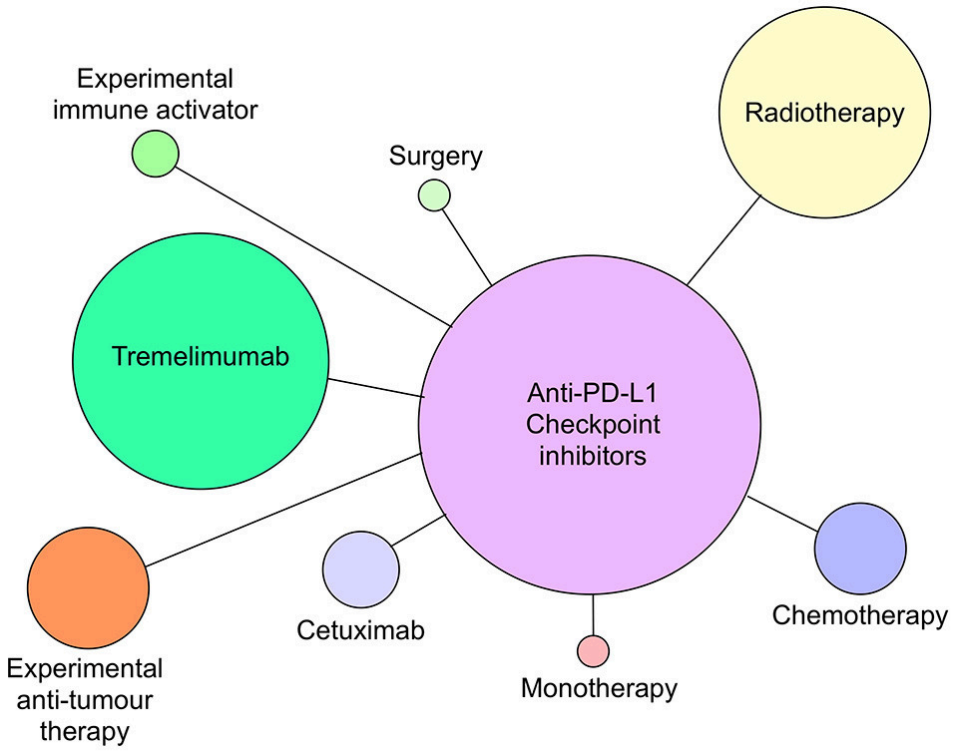
Anti-PD-L1 antibodies atezolizumab, avelumab and durvalumab currently in clinical trials as monotherapy or combined with radiotherapy, chemotherapy, cetuximab, tremelimumab (anti-CTLA-4), surgery, or novel immune activating or tumor suppressive therapies as of July 2018. Circle size reflects relative number of times each type of therapy has been combined with anti-PD-L1 therapy.

Although all of the current six CI therapies can be used as single agent therapeutics, the emphasis now is on identifying combinations of CI therapy or CI therapy with other traditional therapies that will increase both anti-tumor efficacy and patient response frequency. Since 2015, many combination therapies have increased patient responses compared with single CI therapies alone. In metastatic melanoma, combination of nivolumab and ipilimumab (87) were significantly more effective in generating productive shrinkage of tumors in a higher frequency of patients than either of the CI therapeutics alone. For HNSCC, there are >100 clinical trials registered and most of those are combination therapies (Figures 4, 5). The predominant partner therapy for anti-PD-1 and PD-L1 antibody therapies is radiotherapy in either stereotactic body or intensity modulated forms, which is often further supported by established chemotherapy. Radiotherapy seems a particularly good partner for CI therapies, because it can expose tumor-associated neoantigens that in turn can induce the nascent effector T cell responses that develop under cover of checkpoint blockade (88, 89). With regard to surgery followed by radiotherapy and standard chemotherapy, there is an interesting dichotomy of how checkpoint inhibitors have been combined in current clinical trials. While one strategy is to administer either anti-PD-1 (pembrolizumab) or PD-L1 (durvalumab) antibodies in the weeks prior to resection, another strategy is to administer these checkpoint inhibitors just after surgery. Presumably, the strategy of administration prior to surgery is based on the notion that pre-treatment will prime an anti-tumor immunity to enable the immune system to clear any residual tumor cells missed during the surgical procedure. Anti-PD-L1 antibody, durvalumab, is notable for its entry into several HNSCC clinical trials together with tremelimumab, the anti-CTLA-4 mAb (see Figure 5).

Anti-PD-1 and PD-L1 antibodies are also in clinical trials in combination with a wide range of experimental treatments that can be broadly divided into therapies that either target and activate host immunity or target and impair tumor survival. Inhibitors that target the enzyme indoleamine 2,3 dioxygenase (IDO) (90, 91), which is used by regulatory immune cells to deplete tryptophan availability to effector T cells, are particularly notable in these experimental combination therapies. In a similar vein, Toll-like receptor agonists, receptive to nucleic acids are also well-represented. Recent evidence suggests the endoplasmic reticulum associated DNA sensor stimulator of interferon genes (STING) to be a key player in a pathway to sense cytosolic nucleic acids (92, 93) and reverse tumor immunosuppression (94). DC activation through the STING pathway can promote tumor rejection after conventional cancer therapies such as radiation therapy (95). In preclinical studies, STING agonists have been shown to be effective alone or in combination with PD-1/PD-L1 blockade, particularly with established tumors that are refractory to checkpoint blockade alone (96, 97). Signalling cascade inhibitors, e.g., PI3K inhibitors (98, 99) or novel tumor associated peptides are examples of therapies that target tumor cells (100).

CI therapies in combination with other immune activating therapies offer the potential to increase response rates for a range of cancers including HNSCC, but there will also be intense focus on immune related adverse events, especially with regard to both bystander damage of otherwise healthy tissues and to local peritumoral tissues. This will be particularly important for HNSCC given the delicacy of many of the structures within and surrounding the oral cavity and oropharynx. Analysis of safety in patients with renal cell carcinoma that received different combination doses of nivolumab and ipilimumab indicated that the frequency of treatment related adverse effects were ubiquitous but manageable (101). Very few of the adverse events, however, were specific to the tumor site and were typically general, e.g., diarrhea and pyrexia (101).

## Other CI therapies

CTLA-4 and PD-1 are not the only immunoregulatory receptors associated with anti-tumor T cell immunity. There are several more checkpoint proteins under investigation, which may have direct therapeutic use or might be used to improve patient stratification and prognosis. These can be divided into two categories—immunosuppressive and immunostimulatory receptors with examples of the former being Lag-3, Tim3, TIGIT, BTLA and VISTA, and of the latter 4-1BB (CD137), OX40 (CD134), ICOS, and CD40. The expression of these receptors on tumor cells, myeloid, and lymphoid immune cells is variable and tumor dependent, and it is likely that some of them will find future therapeutic value as CI therapies (9).

## Modifying current CI therapies

The development of novel CI therapies over the next few years will continue to be an important focus and a critical aspect for future improvement is to fully elucidate how checkpoint inhibitor therapies are functioning at a molecular level. As a corollary to improving CI therapies, identifying mechanisms that will also condition T cells to respond consistently and effectively following CI therapy is also paramount. Much of this work involves identifying next generation vaccines or mechanisms to shape effector T cell phenotypes with potent anti-tumor activity. Both of these strategies will lead to higher patient response frequencies, better safety and hopefully an enduring immunity in most patients.

A generally less studied aspect of CI receptors is that of the functional effects that their soluble counterparts may have on therapeutic outcomes. These alternate receptor isoforms are either actively secreted by the cell or in some cases cleaved off the cell surface to exert their effects (102–105). Both CTLA-4 and PD-1 have soluble counterparts that are produced by alternative splicing of each gene during translation and are therefore under transcriptional control of the cell that expresses them. Soluble CTLA-4 (sCTLA-4) is produced from the omission of exon 3, encoding the transmembrane domain during alternative splicing of the CTLA-4 gene (46). In addition, a frame shift during splicing of exon 2 to 3 gives rise to a unique C terminal amino acid sequence that replaces the cytoplasmic domain of the CTLA-4 receptor. The soluble isoform of PD-1 (sPD-1) is also produced by omission of exon 3 during alternative splicing. These soluble isoforms may be useful as response biomarkers for patients receiving CI therapy but may also impinge upon the therapy itself.

Soluble CTLA-4 is produced by Treg, but also resting T cells, monocytes, B cells and is capable of binding B7.1, B7.2 and B7-H2 (ICOS-L) on APC (106). This secretable isoform can also be produced by some non-immune cells such as pituitary cells (107). Analysis of several autoimmune diseases originally identified high serum levels of sCTLA-4 compared with healthy donors raising the notion that this isoform actively contributes in some way to immune regulation. Indeed, selective antibody blockade of sCTLA-4 enhanced antigen-specific T cell responses *in vitro* significantly increasing cell proliferation and effector cytokine production compared with isotype or anti-CTLA-4 antibodies. Further, in the diffuse B16F10 murine model of metastatic melanoma, selective blockade had reduced the number of tumor lesions comparably with conventional anti-CTLA-4 antibody treatment (106).

The CTLA-4 receptor exists on cell surfaces as a dimer but the dimerizing cysteine residue at position 122 of the receptor isoform is lost during alternative splicing, which has led to the assumption that sCTLA-4 is secreted as a monomer and therefore has less potency that its dimeric cell-bound counterpart. However, another cysteine is present in the C terminal unique amino acid sequence of sCTLA-4 raising the possibility that sCTLA-4 may be as functionally relevant as the receptor isoform in terms of immune regulation.

Does sCTLA-4, therefore, have any effect on current anti-CTLA-4 based CI therapy? A recent retrospective study of melanoma patient responses to ipilimumab CI therapy demonstrated that patients with relatively high serum levels of sCTLA-4 were more likely to respond to ipilimumab treatment compared to individuals with low or absent serum levels (108). Indeed, selective antibody blockade of sCTLA-4 enhanced antigen-specific T cell responses *in vitro* significantly increasing cell proliferation and effector cytokine production compared with isotype or anti-CTLA-4 antibodies (109). These studies suggest that measuring sCTLA-4 serum levels may be a useful biomarker to stratify patients most likely to respond to therapy and even hint that sCTLA-4 may form a target for therapy. Indeed, analyses of CTLA-4 and sCTLA-4 in cancer cells lines suggest that some tumors may use either or both isoforms as part of a previously overlooked immune evasion strategy (110). Several cancer cell lines and tumor sections have been identified to express CTLA-4 with some evidence that some may also be able to produce sCTLA-4 to suppress effector T cell responses (64). In a seminal analysis of primary melanoma cell lines, Laurent et al. identified some cell lines to express and secrete sCTLA-4 (110). Relatively high levels of sCTLA-4 could also be detected in the sera and pleural effusions of mesothelioma patients, suggesting that it may be contributing to some aspect of immune regulation (111). One hypothesis is that tumor cells or induced Treg secrete sCTLA-4 within the local tumor milieu to suppress effector anti-tumor T cell responses. Even if sCTLA-4 has no functional activity at all, anti-CTLA-4 antibodies will bind sCTLA-4, which over time could reduce the amount of antibody available to target the receptor isoform. Although there is evidence of exosome production of PD-L1 as an immunosuppressive mechanism in HNSCC (112), the role that soluble isoforms of CI receptors play in HNSCC is still largely unexplored.

## Conclusions

Checkpoint inhibitor antibodies for the treatment of HNSCC have demonstrated clear benefits in terms of patients' survival and durability of response but can also induce serious immune related adverse events coupled with an inability to consistently and accurately identify patients likely to respond to this type of therapy. The focus now must be on understanding the genetic signatures most likely to be associated with a productive response to CI therapy, while augmenting current therapies to improve their reliability. Soluble isoforms of CI receptors must also be factored to account for any immunoregulatory role or impact on current therapy that they might have.

## Author contributions

All authors contributed areas of their expertise to this review. FW: CTLA-4 and sCTLA-4; LD: soluble isoforms in disease; RA-E: pathology and insights into anti-tumor immunity in HNSCC.

### Conflict of interest statement

FW and LD are shareholders in Aperio Pharma Ltd., a spin-out company currently developing a novel checkpoint inhibitor antibody. The remaining author declares that the research was conducted in the absence of any commercial or financial relationships that could be construed as a potential conflict of interest.
